# UGT85A84 Catalyzes the Glycosylation of Aromatic Monoterpenes in *Osmanthus fragrans* Lour. Flowers

**DOI:** 10.3389/fpls.2019.01376

**Published:** 2019-11-26

**Authors:** Riru Zheng, Zhenyin Zhu, Yanli Wang, Shiyang Hu, Wan Xi, Wei Xiao, Xiaolu Qu, Linlin Zhong, Qiang Fu, Caiyun Wang

**Affiliations:** ^1^Key Laboratory for Biology of Horticultural Plants, Ministry of Education, College of Horticulture and Forestry Sciences, Huazhong Agricultural University, Wuhan, China; ^2^Key Laboratory of Urban Agriculture in Central China, Ministry of Agriculture, Wuhan, China

**Keywords:** linalool, linalool oxide, *Osmanthus fragrans* Lour., glycosylation, UDP-glycosyltransferase, aroma

## Abstract

The monoterpenes linalool and its oxides are the key aroma-active compounds in *Osmanthus fragrans* Lour. flowers. The glycosides of these monoterpenes accumulate throughout flowering, leading to considerable storage of potential aroma constituents that account for the majority of non-volatile aroma compounds. However, the UDP-glycosyltransferase (UGT) responsible for the glycosylation of linalool and its oxides has not been clarified. Four candidate *OfUGTs* (*UGT85A82*, *UGT85A83*, *UGT85AF3*, and *UGT85A84*) with high homology to the known terpenoid UGTs were screened by transcriptome sequencing. Over-expression of the candidate *OfUGTs* in tobacco showed that UGT85A84 glycosylated linalool oxides *in planta*. Since the transcript levels of *UGT85A84* were positively correlated with glycoside accumulation, the recombinant UGT85A84 protein was subjected to reactions with aglycones and sugar donors. Two formate adducts were exclusively detected in UDP-Glc with linalool and linalool oxide reactions by liquid chromatography-mass spectrometry (LC-MS), indicating that UDP-Glc was the specific sugar donor. The kinetic parameters demonstrated that UGT85A84 glycosylated both linalool and lianlool oxides *in vitro*. Further analysis demonstrated that the transcription levels of MEP pathway genes might play an important role in mediating terpenoid glycosylation. Our findings unraveled the mechanism underlying the glycosylation of essential aroma compounds in flowers. This study will facilitate the application of potential aroma contributors in future industries.

## Introduction

*Osmanthus fragrans* Lour., one of the most popular ornamental plants in China, is particularly appreciated for its unique and pleasant aroma (Zheng et al., 2017). The aroma compounds in flowers not only play important roles in attracting pollinators and promoting defense, but also largely determine the commercial value of the flowers. The *O. fragrans* flowers are widely utilized as ingredients in tea, food, wine, and cosmetic products due to the strong aroma (**Figure S8D**). Aroma compounds have been extensively studied for decades. More than 70 volatiles have been identified to date, including terpenoids, benzenoids and their derivatives, and esters (Zeng et al., 2016). Among all the volatiles, aroma-active compounds are the essential contributors to the olfactory features of flowers. Previous studies have shown that terpenoids, such as linalool, ionone, ocimene, and their derivatives are the key characteristic aroma-active compounds in both natural fresh flowers and in artificial industrial products (Wang et al., 2009; Cai et al., 2014). Ionone and its derivatives are abundantly accumulated only in some cultivars of *O. fragrans* flowers (Zeng et al., 2016); while β-Ocimene is widely present in fresh flowers it is rarely detected in flower products. However, linalool and its oxides account for a large proportion of aroma-active compounds in all *O. fragrans* cultivars. In addition, they impart floral and citrus notes to the flowers and corresponding products. Therefore, they are identified as the principle desirable aroma compounds (Cai et al., 2014).

Linalool, as a typical monoterpene, is derived from the plastidial 2C-methyl-D-erythritol 4-phosphate (MEP) pathway (Jozwiak et al., 2017) and undergoes a cascade of oxidation, cyclization, acylation, hydroxylation, and/or methylation reactions to produce a variety of oxides, alcohols, and ketones (Boachon et al., 2015). A fraction of volatile aroma compounds is released, while the rest are stored as free components or are further transformed into glycosylated conjugates (Lucker et al., 2001; Friedericke et al., 2014a). Glycosylated conjugates are generally more abundant than volatile and free aglycones in many fruits, and they tend to be liberated during product processing, making them potential contributors to aroma quality (Tina et al., 2016). Moreover, previous studies have demonstrated a ripening-related pattern of glycoside accumulation in some fruits and flowers (Yar-Khing et al., 2014; Song et al., 2016; D’;Onofrio et al., 2017). Our previous study revealed that in *O. fragrans* flowers, the emission of volatile linalool and its oxides increased significantly from the initial to full blossoming stage, followed by a subsequent decrease at the late blossoming stage. However, the accumulation of free and glycosylated conjugates did not follow this pattern. Glycosylated 8-hydroxylinalool and linalool oxide showed continuous increase throughout the whole blossoming stage, and more than 75% of non-volatile linalool derivatives were converted into glycosides at the late blossoming stage (Zeng et al., 2016), suggesting that there were common mechanisms for the glycosylation of aroma compounds (Yar-Khing et al., 2014).

Glycosylation reactions are central to numerous biological processes. In general, glycosylation involves sugar polymerization or sugar conjunction with other biomolecules such as proteins, lipids, nucleic acids, and other small molecules (Tiwari et al., 2016). The addition of sugar donors to these aglycones can change their solubility, chemical properties, compartmentation, biological activity, and membrane transportation (Yonekura-Sakakibara and Hanada, 2001). Uridine diphosphate glycosyltransferases (UGTs, EC 2.4.x.y) are the key enzymes catalyzing glycosylation, and they are widely present in plants (Song et al., 2018). All known UGT enzymes contain a universal motif of 44 amino acids (Caputil et al., 2012). They can utilize a variety of sugar donors including UDP-glucose, UDP-xylose, UDP-rhamnose, and UDP-galactose. However, UGTs often show a preference for specific sugar donors (Jin et al., 2013; Shoji et al., 2015). Although the UGTs could catalyze the reactions between sugar donors and a wide range of substrates, the terpenoid UGT sequences are comparatively conserved. For example, AdGT4 from grape (*Vitis vinefera*), which catalyzes terpenes and primary alcohols, shows high amino acid homology to the iridoid-specific UGT85A42 from *Gardenia jasminoides* (Nagatoshi et al., 2011; Yar-Khing et al., 2014) and a cyanohydrin mandelonitrile-specific UGT85A19 from *Prunus dulcis* (Franks et al., 2008). Therefore, an effective strategy to identify candidate terpenoid UGT genes is to compare the current transcriptome profiling with the known UGT sequences in arabidopsis (*Arabidopsis thaliana*), grape (*Vitis vinifera*), tea (*Camellia sinensis*), kiwi (*Actinidia deliciosa*), and other plants (Yar-Khing et al., 2014; Friedericke et al., 2014b; Shoji et al., 2015).

In the present study, we conducted the transcriptome sequencing and identification of differentially expressed genes (DEGs) in four blossoming stages of *O. fragrans* flowers to select putative *OfUGT* genes involved in the glycosylation of linalool and its oxides. We also investigated possible relations between gene expression and aroma compound glycosylation. The candidate *OfUGT* gene was subjected to further functional characterization. Previous studies of UGTs mainly focused on fruits and leaves respectively used for wine and tea. Flowers have become important ingredients in food and health industries. However, the mechanism underlying aroma compound glycosylation in flowers remains unclear. Our findings elucidate the glycosylation of crucial aroma compounds in flowers. This study provides new information for improving aroma quality in ornamental plants.

## Materials and Methods

### Plant Materials

*O. fragrans* ‘Liuye Jingui’ was cultivated on the campus of Huazhong Agricultural University in Wuhan, China. Flowers were all harvested at 09:00 in October. The blossoming stage of a single flower lasts for approximate 7 days and is divided into four stages: bud, initial blossoming stage, full blossoming stage, and late blossoming stage (**Figure S8B**). The duration of the full blossoming stage was relatively stable and the flowers from this stage were collected at 06:00, 12:00, 18:00, and 00:00 h for 3 successive days for analysis of *OfUGT* expression. Flower samples were frozen in liquid nitrogen and stored at –80°C for transcriptome sequencing and qRT-PCR determination. Each experiment included three biological replicates.

### Library Preparation for Transcriptome Sequencing

RNA extraction was carried out as previously described (Zeng et al., 2016) and qualified RNA samples, which had been quality-checked by nanodrop, were sent to Novogene Bioinformatics Technology Co. Ltd (Beijing) for transcriptome sequencing. 1.5 µg RNA per sample was used as input material. Sequencing libraries were generated using NEBNext^®^ Ultra™ RNA Library Prep Kit for Illumina^®^ (NEB, USA) following the manufacturer’s recommendation, and index codes were added to distinguish sequences from each sample. The library quality was assessed in the Agilent Bioanalyzer 2100 system. The clustering of the index-coded samples was performed in a cBot Cluster Generation System using TruSeq PE Cluster Kit v3-cBot-HS (Illumia) according to the manufacturer’s instruction. The clusters were sequenced on an Illumina Hi-seq platform and paired-end reads were generated. A total of approximately 50 million raw reads were sequenced in the library. After the removal of the reads with adapters, poly-N, low-quality reads, and clean reads were obtained for further analysis (Li and Dewey, 2011). The base error rate was 0.01%–0.02% and the average Q20 and Q30 were higher than 97% and 92%, respectively. In addition, the average GC content was higher than 43%, suggesting that this Illumina sequencing was of high quality (**Table S1**). The SRA accession number of the transcriptome sequencing is PRJNA564449 in the NCBI database.

### Transcriptome Assembly and Annotation

Gene function was annotated based on the databases (**Supplementary Table S3**): National Center for Biotechnology Information (NCBI: http://www.ncbi.nlm.nih.gov/), Pfam (http://pfam.sanger.ac.uk), GO (http://geneontology.org/) (**Figure S1**), KOG/COG (http://www.ncbi.nlm.nih.gov/COG/) (**Figure S2**), Swiss-Prot (https://www.ebi.ac.uk/uniprot), and KEGG (https://www.kegg.jp/) (**Figure S3**).

### Differential Expression Analysis and Enrichment Analysis

Gene expression levels of each sample were estimated by RSEM software (Li and Dewey, 2011). Differential expression analysis was performed using the DESeq R package (1.10.1), which provides the data for determining differentially expressed genes (DEGs) using a model based on the negative binomial distribution. The resulting P values were adjusted in the Benjamini and Hochberg’s approach for controlling the false discovery rate (Wang et al., 2010). DEGs were designated as those with an adjusted P value <0.05. GO enrichment analysis of DEGs was implemented by the GO seq R packages based on Wallenius non-central hyper-geometric distribution, which can adjust the gene length bias of DEGs. We used KOBAS software to test the statistical enrichment of DEGs in KEGG pathways (Wang et al., 2010).

### Quantitative Real-Time PCR Analysis

Quantitative real-time PCR (qRT-PCR) was conducted to quantify gene expressions. The RNA samples used for qRT-PCR were the same as with those used for the transcriptome sequencing. The experiments were performed on Applied Biosystems 7500 Fast Real-Time PCR platform with the SYBR^®^ Premix Ex Taq™ II mix (Takara Biotechnology Co., Ltd, Dalian, Japan), and the results were analyzed by the Applied Biosystems 7500 software (Applied Biosystems Life Technologies). The results of triplicate assays were then calculated by the 2^-ΔΔCt^ method. The primers for qRT-PCR analysis are listed in **Table S2**.

### Cloning and Sequence Analysis of *OfUGT* Genes

**Figure 1:**
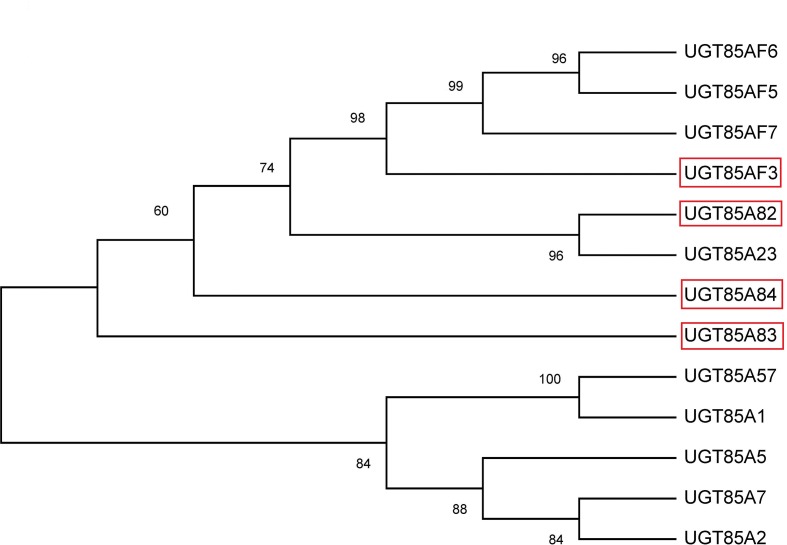
Phylogenetic analysis of the four candidate terpenoid OfUGTs with other plant terpenoid UGTs. The phylogenetic tree was constructed according to the Maximum Likelihood method by MEGA 6.1 software.

The full lengths of four *OfUGT* genes were obtained by the rapid amplification of cDNA ends (RACE) method using the original sequences from the transcriptome sequencing. The 5’ and 3’-RACE-Ready cDNAs were synthesized separately using the BD SMARTER™ RACE cDNA Amplification Kit (Clontech, Mountain View, CA, USA). The amplified sequences were cloned into pEASY-T1 vectors (TransGene Biotech Co., LTD, Beijing, China) and three independent clones were sequenced to eliminate PCR errors. The open reading frames (ORFs) were predicted by the NCBI ORF Finder (https://www.ncbi.nlm.nih.gov/orffinder/). All primers applied are listed in the **Table S2**. A phylogenetic tree was constructed with other reported terpenoid UGTs in the method of Maximum Likelihood by MEGA 6.1 software (**Figure 1**). The information of the reported terpenoid UGTs was described in **Table S4** and the amino acid alignment between OfUGTs and other terpenoid UGTs with high homology was demonstrated in **Figure S5**.

### Transient Expression of *OfUGT* Genes

The control and over-expression constructs were prepared as reported by Zeng et al. (2016). These plasmids were transformed into *Agrobacterium tumefaciens* strain EHA105 by electroporation. Transient expression experiments with *Nicotiana benthamiana* leaves were performed as described by Zeng et al. (2016). The glycosides of linalool and its oxides were not detected in *N. benthamiana* leaves without exogenous treatment. Five days after the inoculation, the leaves were infiltrated with different aglycone solutions (50 µM linalool and 200 µM linalool oxides in 0.5 M MES + 1 M MgCl_2_). Four hours after infiltration, the leaves were detached and frozen at –80°C for glycoside determination (Yar-Khing et al., 2014). Glycoside extraction coupled with GC-MS was performed to quantify the glycoside contents according to the previously described methods (Zheng et al., 2017).

### Heterologous Expression of Recombinant UGT85A84 Protein

The full-length *UGT85A84* ORF was amplified and cloned into the pET21b and pET15b expression vectors (Novagen) using the primers listed in **Table S2**. The two plasmids were co-transformed into *Escherichia. coli* BL21 (DE3) pLysS cells (Promega, Madison, WI, USA). The co-transformed cells were cultured in lysogeny broth medium supplemented with 100 µg/ml ampicillin at 37°C until OD_600_ approached 1.0–1.2. The temperature was then shifted to 16°C, and the cells were incubated with 0.2 mM isopropyl β-D-1-thiogalactopyranoside for 16 h. Subsequently, the cells were harvested by centrifugation, resuspended in lysis buffer (pH 8.0) containing 25 mM Tris-HCl and 150 mM NaCl and lysed by a JN-02 homogenizer. After centrifugation, the supernatant was loaded onto a column equipped with Ni^2+^ affinity resin (Ni-NTA, Qi- agen), subsequently washed with a buffer (pH 8.0) containing 25 mM Tris-HCl, 150 mM NaCl, and 15 mM imidazole and eluted with a buffer (pH 8.0) containing 25 mM Tris-HCl and 250 mM imidazole. The eluted protein was further purified by anion-exchange chromatography (Source 15Q 10/100, GE Healthcare) with a linear NaCl gradient in the buffer (pH 8.0) containing 25mM Tris-HCl, 1M NaCl, and 2mM dithiothreitol. Then, the elution peak was concentrated to 1 ml and subjected to size-exclusion chromatography (Superdex-200 Increase 10/300, GE Healthcare) in the buffer (pH 8.0) containing 25mM Tris-HCl, 150mM NaCl, and 5mM dithiothreitol (DTT). The purity of the protein was analyzed by sodium dodecyl sulfate polyacrylamide gel electrophoresis (SDS-PAGE) and the protein was quantified using a NanoDrop 2000 spectrophotometer (Thermo Scientific, USA).

### OfUGT85A84 Enzyme Assay

Enzymatic activity assays were carried out according to the description by Shoji et al. (2015) with modification. The enzymatic reaction mixture (200 µl) consisted of 0.5 mM aglycone (linalool or linalool oxide), 0.5 mM sugar donor (UDP-glucose, UDP-rhamnose or UDP-xylose), 100 mM Tris-HCl buffer (pH 7.5, 10% (v/v) glycerol and 10 mM 2-morcaptoethanol), and 5 µg purified protein. After incubation of the mixture at 37°C for 16 h, the reaction was terminated by the addition of 1 µl of 24% trichloroacetic acid (TCA). Except for the concentrations of substrates, the same assay conditions were used for measuring the UGT85A84 kinetic parameters. To determine the kinetic parameters of linalool and linalool oxide, the concentration of aglycones was set within the range from 5 µM to 50 mM (linalool) or to 5mM (linalool oxide), with a constant UDP-glucose concentration of 0.5 mM. To determine the kinetic parameters of UDP-glucose, UDP-glucose concentration was set from 25 µM to 10 mM with a constant linalool or linalool oxide concentration of 0.5 mM. All the reactions were incubated at 37°C for 1 h and repeated in triplicate. The reaction products were subjected to LC-MS analysis. The apparent *K*_m_ and *k* *_cat_* values for each aglycone and sugar donor were determined by Michaelis-Menten fitting in OriginPro 9.1 software.

LC-MS analysis was performed in the Thermo Scientific^™^ UltiMate^™^ 3000RS system. The separation was conducted on a Thermo Scientific^™^ Syncronis C18 column (100*2.1 mm, 1.7µm) at 40°C. Mobile phases consisted of phase A referring to water with 0.1% formic acid and phase B referring to methanol with 0.1% formic acid. Chromatographic separation was achieved by gradient elution at a flow rate of 0.3 ml/min. The samples were eluted with the following linear gradient: 0–1min, 2% B; 1–5min, 2-95% B; 5–7min, 95% B; 7–7.1, 95–2% B; 7.1–9, 2% B. The MS analysis was performed on a Thermo Scientific^™^ Q Exactive Plus hybrid quadrupole-Orbitrap benchtop high-resolution mass spectrometer equipped with heated electrospray ionization. Sheath gas was set at 40 arbitrary units, auxiliary gas at 15 arbitrary units, and spray voltage at 3,800 V for positive ionization and 3,500 V for negative ionization. Identification of target compounds was carried out using Data-Dependent Acquisition (DDA). The HCD collision energy was set at NCE 20 for negative mode and was set at NCE 30 50 80 for positive mode. Quantification was performed in the full scan negative mode.

### Statistical Analysis

The data were expressed as the mean values from three independent biological replicates, with error bars indicating the standard error mean (SEM). Significance was determined using a one-way analysis of variance. For differences between groups, the least significant difference (LSD) *t*-test was employed. The relative coefficients were analyzed by Pearson in SPSS.

## Results

### Four *OfUGTs* Were Selected as Candidate *UGT*s for Linalool and its Oxides

Four candidate *OfUGT* genes (Cluster 9575.22195.0, Cluster 9575.63104, Cluster 9575.37686, and Cluster 9575.35647) were selected for functional characterization based on their high similarity (> 40%) in amino acid sequence to the known terpenoid *UGTs* (Song et al., 2018). The ORF sequences were 1,458 bp, 1,449 bp, 1,422 bp, and 1,434 bp, respectively, and were designated as *UGT85A82*, *UGT85A83, UGT85AF3*, and *UGT85A84* (GenBank accession numbers: MG767214, MG767215, MG767216, and MG767217), respectively. A phylogenetic tree was constructed based on the known terpenoid UGT proteins (**Figure 1**; **Table S4**). A close relationship was observed between UGT85A82 and UGT85A23 from *Catharanthus roseus* (Nagatoshi et al., 2011), with the latter involved in iridoid glycosylation. UGT85A83 and UGT85A84 showed high amino acid identity to the UGT proteins from *A. thaliana* and *Rubus suavissimus*, including UGT85A1, UGT85A2, UGT85A5, UGT85A7, and UGT85A57, whose function was mainly to glycosylate monoterpenes and diterpenoid (Song et al., 2018). UGT85AF3 belonged to UGT85AF subfamily. Only 7 genes were clustered into this subfamily to date (**Table S4**). UGT85AF3 showed significantly high bootstrap value with UGT85AF5, UGT85AF6, and UGT85AF7 (**Figure 1**).

### Massive Carbohydrate Biosynthesis Might Facilitate the Glycosylation of Aroma Compounds Since the Full Blossoming Stage According to Illumina Hiseq mRNA Sequencing

Since the glycosylated linalool and its derivatives showed the maximum increase from the full blossoming stage (Zeng et al., 2016), further DEG analysis was conducted based on the KEGG enrichment (**Figure S4**). In terms of the DEG number, the most substantial differences between the initial and full blossoming stages were found in the categories of ‘hormone signal transduction’, ‘carbohydrate metabolism’, and ‘phenylpropanoid biosynthesis’ (**Figure S4A**). Notably, 37 unigenes involved in starch and sucrose metabolism, 26 unigenes involved in glycolysis, 25 unigenes involved in galactose metabolism, and 14 unigenes involved in fructose and mannose metabolism were up-regulated from the initial to full blossoming stage (**Figure S4A**). In contrast, the unigenes associated with starch, sucrose, and galactose metabolism were significantly down-regulated from the full to late blossoming stage (**Figure S4B**). We therefore hypothesized that the increase in the expressions of genes involved in the primary metabolism facilitated the glycosylation of aroma compounds since the full blossoming stage.

### *UGT85A84* Expression Was Positively Correlated With the Glycosylation of Linalool and Its Oxides

**Figure 2:**
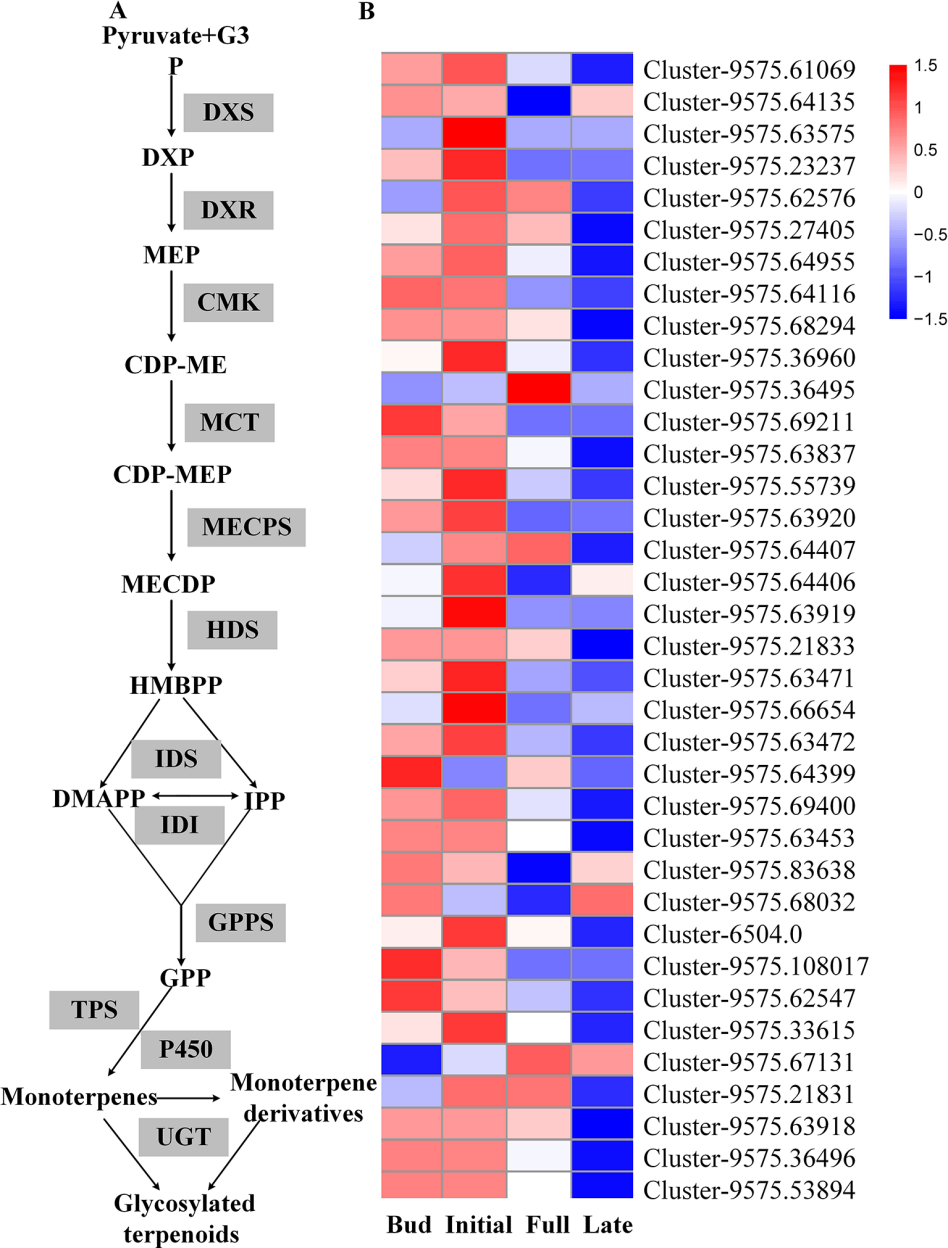
The transcript levels of genes involved in glycosylation of linalool and its oxides during the blossoming stages in *Osmanthus fragrans* flowers according to transcriptome sequencing. **(A)** The biosynthesis pathways of linalool and its oxides glycosides. **(B)** The expression heatmap of MEP pathway genes. The up- and down-regulated genes were demonstrated by red and blue bars, respectively.

**Figure 3:**
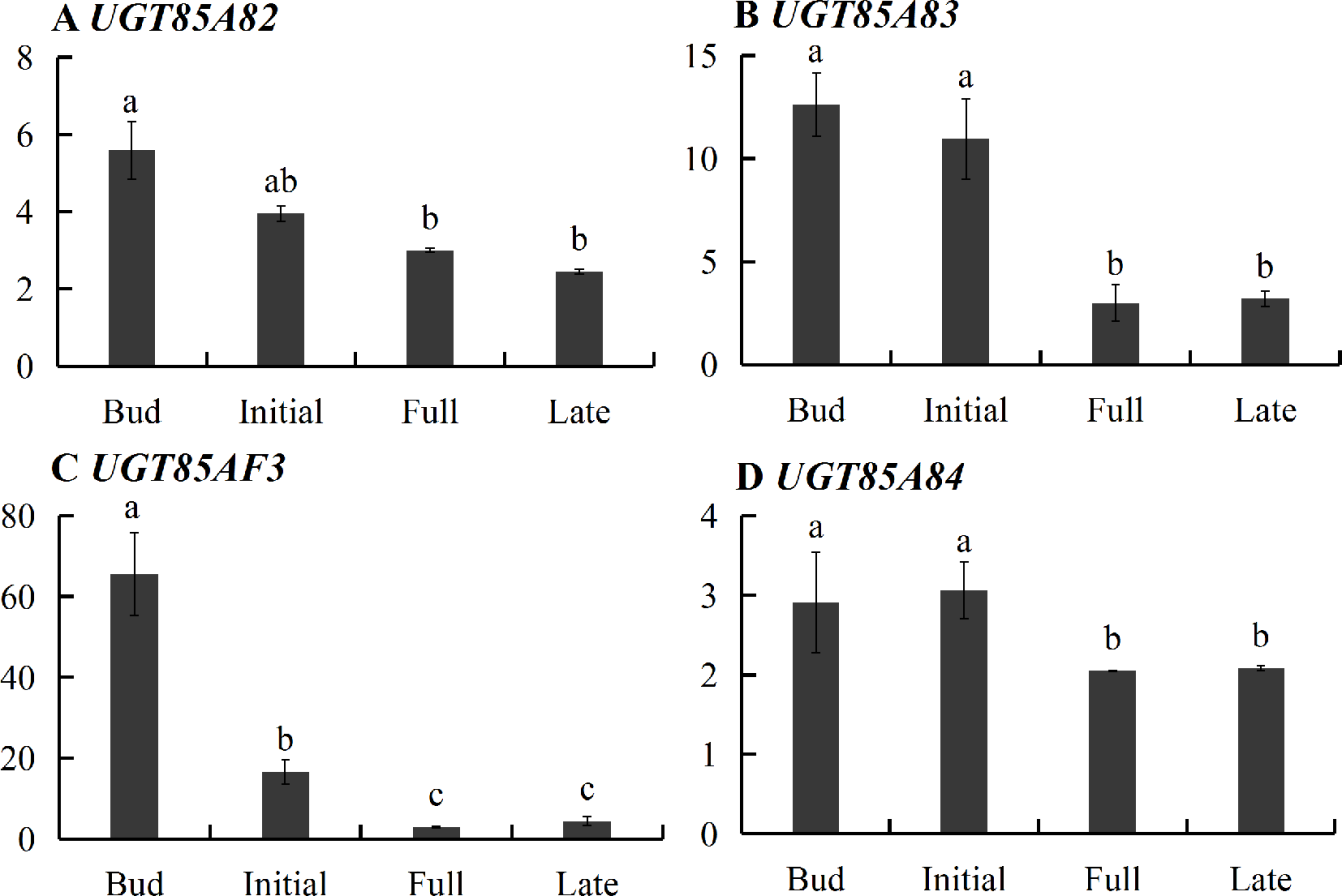
Developmental expression of the four candidate terpenoid *OfUGTs* during the blossoming stages in *Osmanthus fragrans* flowers. The relative expression of the unigenes was calculated by the 2^-ΔΔCt^ method. Data are presented as mean ± standard error of the mean (SEM) (n = 3). Different letters indicate significant difference as determined by one-way ANOVA followed by LSD *t*-test (P 0.05).

Transcriptome sequencing was applied to measure the expressions of genes responsible for linalool and its oxide glycosylation in the four blossoming stages (**Figure 2**). Linalool and its oxides were synthesized through the MEP pathway and the expressions of a series of relevant genes including *DXS*, *DXR*, *CMK*, *MCT*, *MECPS*, *HDS*, *IDS*, *IDI*, and *GPPS*, and terpene synthase genes (*TPSs*) were investigated by transcriptome sequencing (**Figure 2A**). The results showed that the expressions of all the upstream genes were basically increased from bud to initial or full blossoming stage and decreased in the late blossoming stage, directly leading to the massive biosynthesis of linalool and its oxides at the full blossoming stage (**Figure 2B**, **Figure S8A**). Unexpectedly, qRT-PCR results demonstrated that the expressions of all four candidate *OfUGTs* presented a continuously decreasing trend through the blossoming stages (**Figure 3**), which was opposite to the increasing trend of terpenoid glycosides (Zeng et al., 2016). Taken together, despite the low expression of candidate *OfUGTs*, the abundant aglycone and sugar donor supplies facilitated the conversion from volatiles to glycosides following the full blossoming stage (**Figure S8A**).

**Figure 4:**
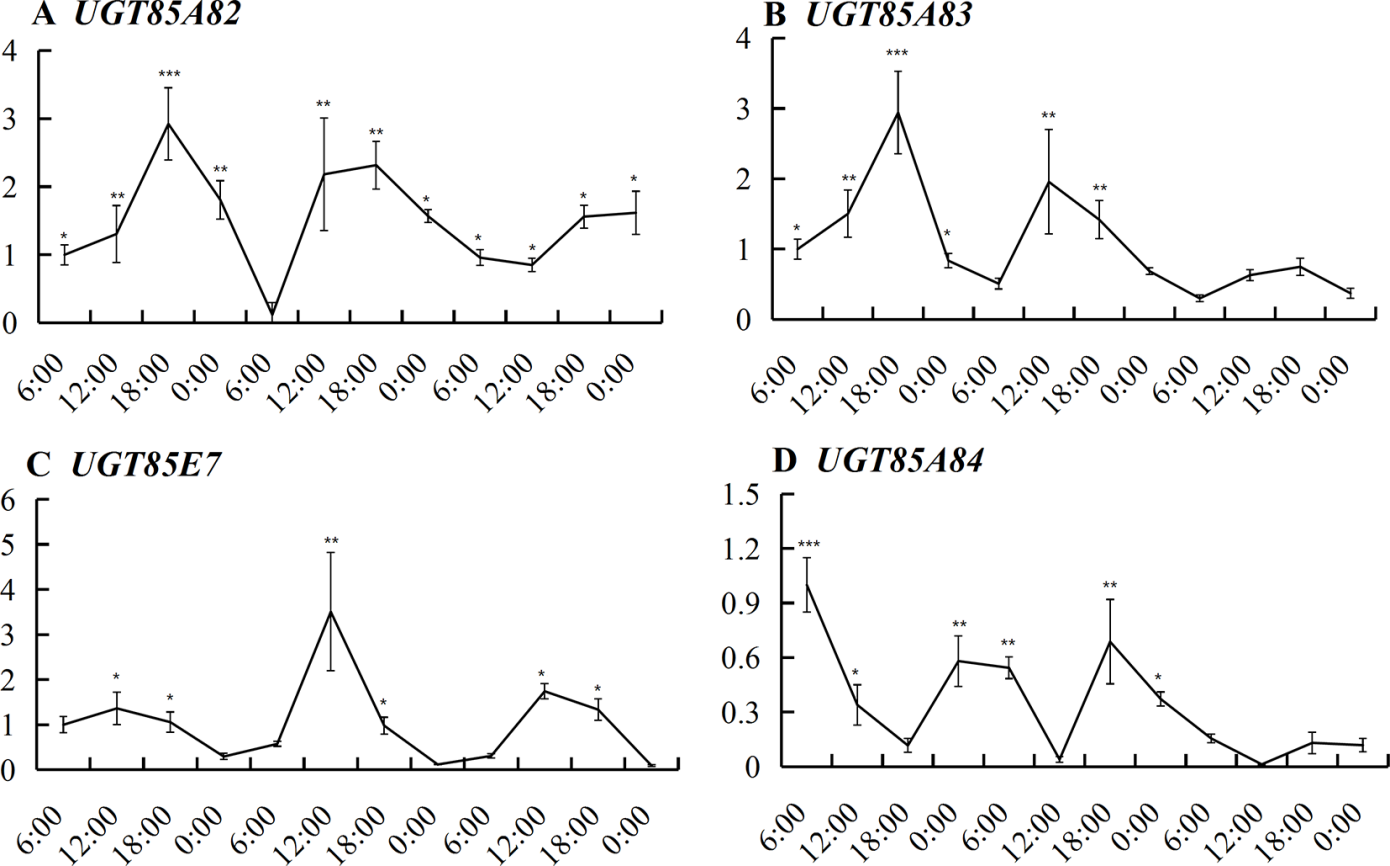
Expression of the four candidate terpenoid *OfUGTs* during the full blossoming stage over a circadian period. Quantitative real time (qRT)-PCR analysis was performed to determine the relative expression. Data are presented as mean ± standard error of the mean (SEM) (n = 3). Different marks indicate significant difference as determined by one-way ANOVA followed by LSD *t*-test (*P 0.05, **P 0.01, ***P 0.001).

*UGT85A82* expression showed obvious circadian rhythm during the full blossoming stage (**Figure 4**). The expressions of four *OfUGTs* simultaneously increased during the day and decreased during the night (**Figure 4**). Further correlation analysis between their transcript levels and three forms of linalool and its oxides were conducted to screen the candidate terpenoid *OfUGTs*. Only *UGT85A84* expression showed significantly positive correlation with the accumulation of glycosides (**Table 1**). MEP pathway genes also exhibited the circadian expression patterns, which was in line with continuous increase of linalool and its oxide glycosides from 06:00–00:00 (Zheng et al., 2017). Further correlation analysis also presented a positive association between MEP pathway gene expressions and glycoside accumulation (**Table S5**).

**Table 1:** The correlation between transcript levels of the four candidate terpenoid *OfUGTs* and the contents of volatile, free, and glycosylated aroma compounds.

Genes	Volatile linalool and its oxides	Free linalool and its oxides	Glycosylated linalool and its oxides
*UGT85A82*	-0.35	-0.59	-0.24
*UGT85A83*	-0.07	0.19	0.23
*UGT85AF3*	0.55	0.59	-0.14
*UGT85A84*	-0.07	0.31	0.70

^1^The relative coefficients were analyzed by the Pearson of SPSS according the circadian transcript levels and aroma compounds during the full blossoming stage of O. fragrans flowers.

### Over-Expression of *UGT85A84* Significantly Increased Linalool Oxide Glycoside *in* *Planta*

**Figure 5:**
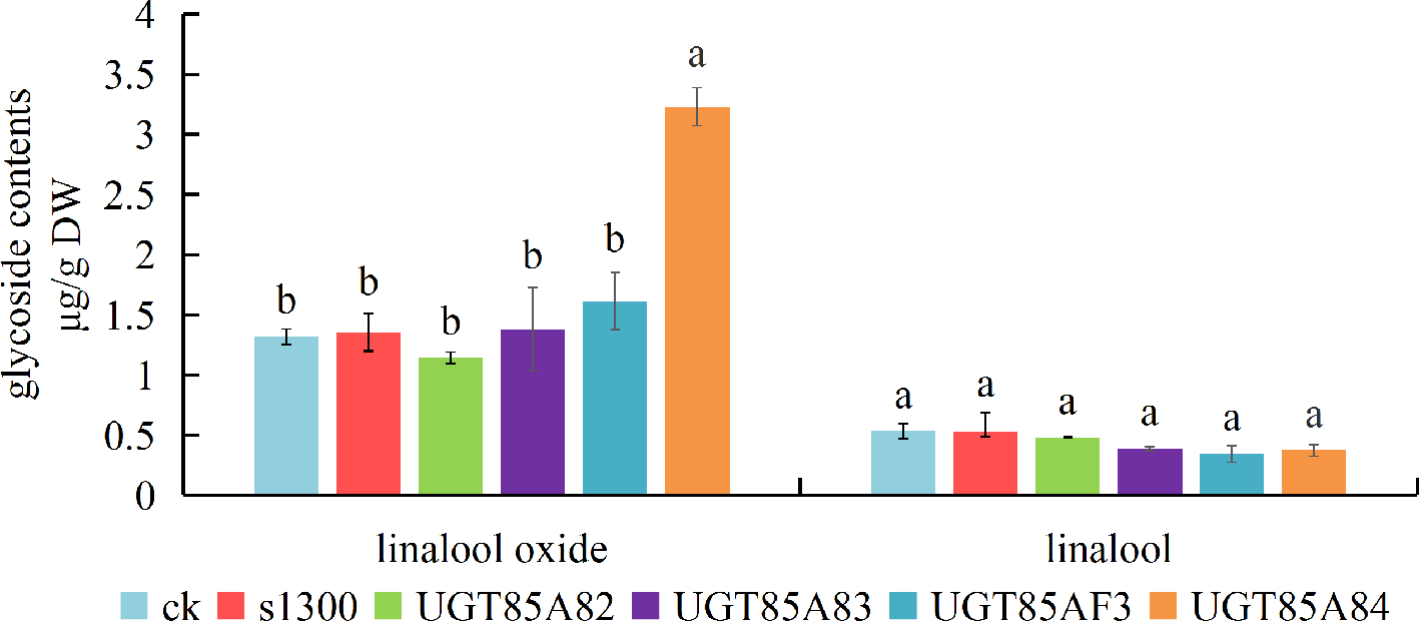
Functional characterization of the four candidate terpenoid *OfUGTs* in *Nicotiana benthamiana* leaves. The *N. benthamiana* leaves were infiltrated with a control construct (S1300-GUS) or S1300-*OfUGTs* in the presence of linalool and its oxides. Purified glycosides were treated with β-glucosidase and the volatiles released were extracted into a solvent for gas chromatography-mass spectrometry (GC-MS) analysis. The glycoside contents were qualified and compared between aglycone-injected plants without transgene (CK) and aglycone injected together with transgenic plants (S1300, *OfUGTs*). Data are presented as mean ± standard error of the mean (SEM) (n = 3). Different letters indicate significant difference as determined by one-way ANOVA followed by LSD *t*-test (P 0.05).

The experiments of transient over-expression coupled with the substrate injection in *N. benthamiana* leaves were conducted to investigate the glycosides produced by candidate *OfUGTs in planta*. The leaves were infiltrated with Super1300-OfUGT or a control construct in the presence of linalool and its oxides. The glycosides were then extracted and purified after incubation. Subsequently, GC-MS analysis calculated the glycoside contents by quantifying the corresponding aglycones released from the glycosides. The results showed that considerable glycosides of linalool and its oxides were accumulated in the leaves with substrate injection (CK), while no glycoside was detected from wild-type tobacco leaves, indicating that the injection effectively promoted the absorption and conversion of the substrates by the leaves. It was worth noting that remarkable increase in glycoside of linalool oxide was only observed in *UGT85A84*-infiltrated plants, suggesting that UGT85A84 was capable of catalyzing linalool oxides. All the four OfUGTs showed low activities towards linalool since no significant increase in linalool glycoside was observed in the transgenic plants (**Figure 5**). This result was similar to the phenomenon in *O. fragrans* flowers. Linalool glycoside was approximately 3 mg/g FW, whereas linalool oxide glycoside was more than 40 mg/g FW at the late blossoming stage (Zeng et al., 2016), suggesting that linalool oxide was more likely to be glycosylated.

### Recombinant UGT85A84 Protein Glycosylated Linalool and Its Oxides Using UDP-Glc *in vitro*

**Figure 6:**
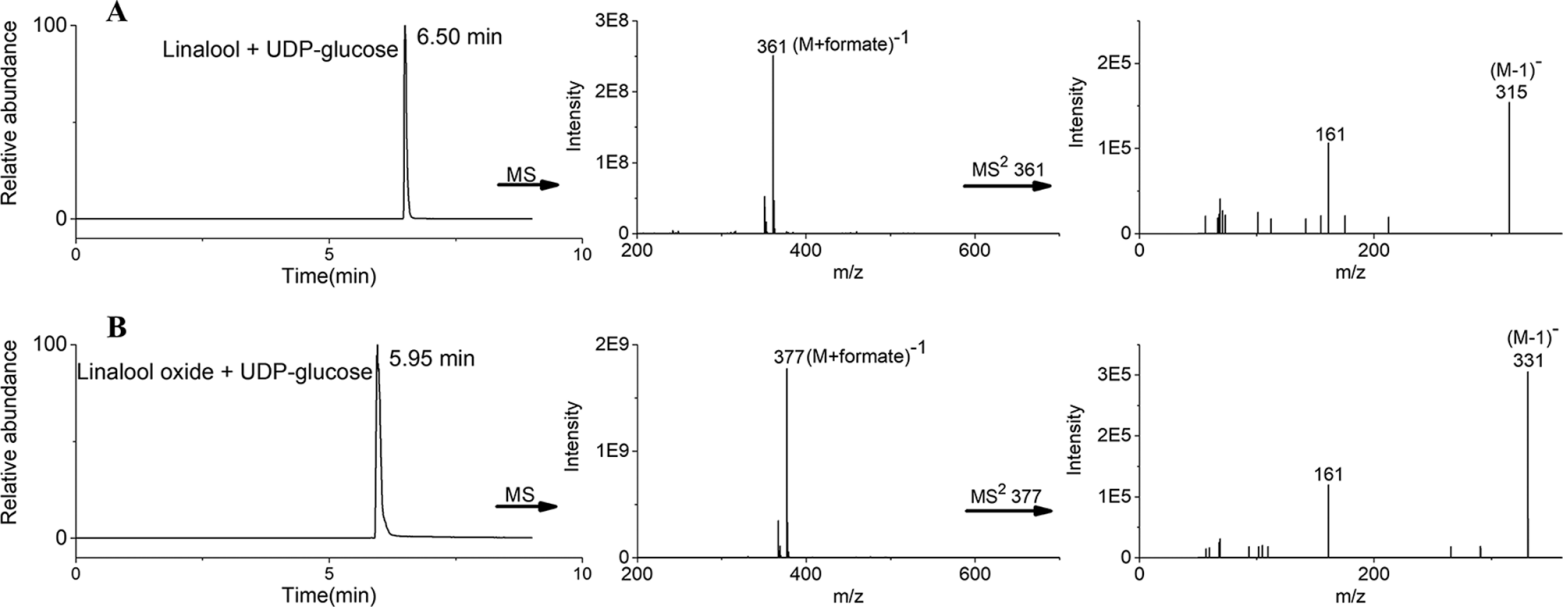
Liquid chromatograph-mass spectrometry (LC-MS) analysis of UGT85A84 reaction products. LC-ESI-MS (Extracted Ion Chromatogram) base peak plots in negative mode for **(A)** linalool + UDP-Glc, **(B)** linalool oxide + UDP-Glc. The full scan and MS^2^ data identified two exclusive formate adducts only in the UDP-Glc reactions. No products were detected in the UDP-Xyl and UDP-Rha reactions. Results from a 16 h incubation were presented.

**Figure 7:**
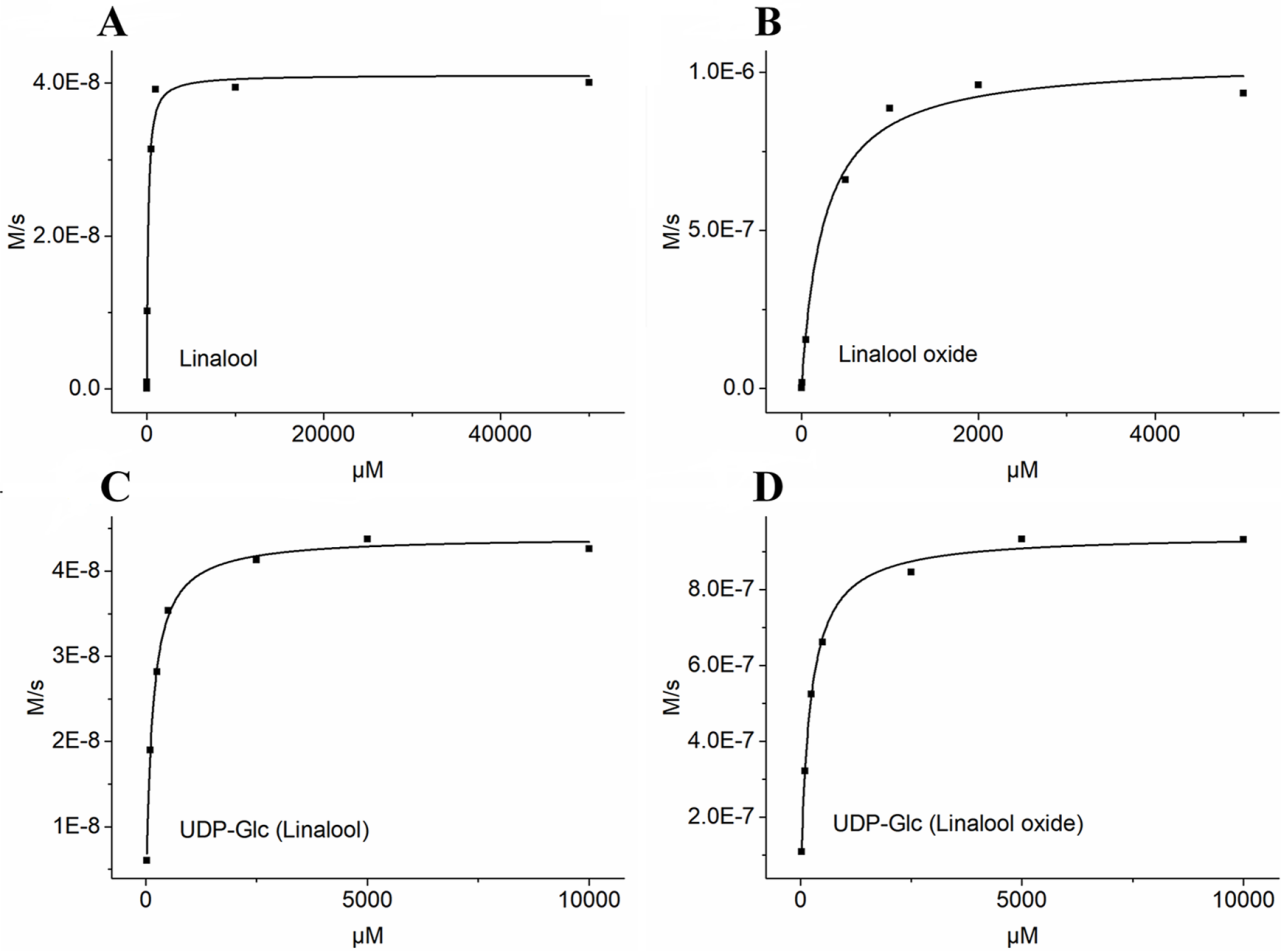
Determination of kinetic parameters of UGT85A84 in linalool + UDP-Glc and linalool oxide + UDP-Glc reactions by the hyperbolic Michaelis-Menten saturation curve. **(A)** UDP-Glc (0.5mM) + linalool (5 µM to 50 mM). **(B)** UDP-Glc (0.5mM) + linalool oxide (5 µM to 5 mM). **(C)** linalool (0.5mM) + UDP-Glc (25 µM to 10 mM). **(D)** linalool oxide (0.5mM) + UDP-Glc (25 µM to 10 mM). The apparent Km and Vmax values for each sugar acceptor and the sugar donor were determined by Michaelis-Menten fitting in OriginPro 9.1 software.

Based on the transcript expression analysis and functional identification *in planta*, *UGT85A84* was selected as the key *OfUGT* and was subjected to further analysis. The recombinant protein of UGT85A84 was expressed in *E. coli* and the purified protein was verified by SDS-PAGE (**Figure S6**). Target analysis of LC-MS was an effective method for determining the specific product (Takafumi et al., 2018). Thus, the biochemical characteristics of UGT85A84 were investigated with different sugar donors (UDP-Glc, UDP-Xyl, UDP-Rha) and aglycones (linalool, linalool oxides), respectively (**Figure S7**). After 1 h or 16 h incubation, only two glycosylated products, with retention time of 6.50 min and 5.95 min, were formed in the UDP-Glc reaction mixtures (**Figure 6**; **Figure S8C**). No products were detected in the UDP-Xyl and UDP-Rha reactions, demonstrating that UGT85A84 had no catalytic activity towards these two sugar donors. Further full scan and MS/MS mass spectral data were used to identify and quantify the reaction products. Linalool-glycoside was shown to be the corresponding formate adduct based on the *m/z* 361 [M + formate]^-1^ in full scan mode and the expected pseudo-molecular ion at *m/z* 315 in MS^2^ (**Figure 6**) in the UDP-Glc + linalool reactions. Linalool oxide-glycoside was the corresponding formate adduct based on the *m/z* 377 [M + formate]^-1^ in full scan mode and the expected pseudo-molecular ion at *m/z* 331 in MS^2^ (**Figure 6**) in the UDP-Glc + linalool oxide reactions. *K*m and *kcat* values were obtained for the linalool and linalool oxide with a constant UDP-Glc concentration (0.5 mM) and for the UDP-Glc with a constant linalool and linalool oxide concentration (0.5 mM) from a hyperbolic Michaelis-Menten saturation curve, respectively (**Figure 7**). The apparent *K*m for linalool and linalool oxide were 136 µM and 202 µM (**Table 2**), respectively, indicating that UGT85A84 had higher catalytic efficiency with linalool than with linalool oxide *in vitro*. This result contrasts the catalytic performance in *N. benthamiana* leaves, which might be explained by the fact that linalool is a low molecular weight compound that is highly volatile and thus might be easily released instead of being glycosylated in *N. benthamiana* leaves. Taken together, the result demonstrated that UGT85A84 was capable of glycosylating linalool and linalool oxide using UDP-Glc as a specific sugar donor.

**Table 2:** Kinetic properties of purified recombinant UGT85A84 enzyme.

Substrate	*K*_m_ (μM)	*k* *_cat_* *(S* *^-1^* *)*	*k* *_cat_*/*K*_m_ (s^-1^ mM^-1^)
linalool	135.712.48	0.09	718
linalool oxide	202.361.63	2.12	10494
UDP-Glc (linalool)	138.3613.38	0.09	666
UDP-Glc (linalool oxide)	248.195.77	2.33	9396

^1^The kinetic parameters of UGT85A84 towards lianlool and linalool oxide as substrates were obtained from substrate concentrations ranging from 25μM to 10 mM with a fixed UDP-Glc concentration of 0.5 mM. The kinetic parameters of UGT85A84 towards UDP-Glc were obtained from substrate concentrations ranging from 25μM to 10 mM with a fixed linalool or linalool oxide concentration of 0.5 mM, respectively. Data are presented as meanstandard error (SEM) (n = 3).

## Discussion

### Potential Function of Glycosylated Linalool and Its Oxides

Volatile aroma compounds have been extensively studied for decades because they not only act as pollinator attractant for better reproduction but also enhance the aroma value of flowers and fruits (Keisuke et al., 2013). Although the odorless non-volatile forms are less concerned compared to the volatiles, the glycosylation of aroma compounds impact the flowers in at least two significant ways. On the one hand, it reduces the abundance of volatiles, resulting in the decreased aroma of fresh flowers; on the other hand, the glycosides can be hydrolyzed during industrial processing, thereby enhancing the aroma of products (Parker et al., 2018). For example, the glycosylated terpenoids in tomato paste are hydrolyzed into their volatile forms during processing and the corresponding volatiles confer a strong aroma (Simona et al., 2009). For another example, most terpenoids are conjugated to sugars in aromatic grapevines and can be a potential source of wine aroma (Bönisch et al., 2014). The predominant volatile aromatic compounds in oolong tea (*C. sinensis*), including linalool, geraniol, and 2-phenylethanol, can only be artificially released during full fermentation because these aroma compounds typically accumulate in the form of water-soluble glycosides in fresh tea leaves (Shoji et al., 2015). As far as *O. fragrans* flowers are concerned, terpenoids are important extracts in essential oils and a large amount of terpenoids can be liberated by β-glucosidase hydrolysis (Wang et al., 2009). Thus, non-volatile terpenoids can serve as significant contributors to aroma in industrial products.

The glycosylation of terpenoids also mediate the balance between pollination and defense. The volatile linalool and its oxides reached the maximum level at the full blossoming stage to attract pollination (Zhang et al., 2013). The cytotoxic volatiles were then converted into harmless glycosylated forms, which were an indirect defense mechanism to protect seeds after blossoming (Raguso, 2016; Song et al., 2018). UGTs were found in the nucleus and cytoplasm to protect the nucleus from toxic damage by excessive terpenoids (Wu et al., 2018).

### UGTs Play Important Roles in Terpenoid Glycosylation

Glycosylation is one of the most ubiquitous modifications of plant secondary metabolites, and plants often contain a large number of UGT genes involved in diverse biological processes. In the current study, we screened four candidate UGT genes responsible for glycosylation of linalool and its oxides. Of them, *UGT85A84* was proved to be capable of glycosylating linalool and its oxide both *in planta* and *in vitro*. *K*_m_ is an important kinetic parameter to reflect the affinity between enzyme and substrate. UGTs often present diverse *K*_m_ values towards different substrates, suggesting that they have a preference for specific substrates (**Supplementary Table S6**). For example, VvGT15b showed significantly higher affinity than other VvUGTs towards terpenoids in grape (Friedericke et al., 2014a; Friedericke et al., 2014b). In this study, the *K*_m_ values of UGT85A84 for linalool and linalool oxide was 135.71µM and 202.36µM, respectively, indicating that UGT85A84 had higher activity towards linalool than linalool oxide. However, UGT85A84 showed better affinity compared to PpUGT85A2 in peach (Wu et al., 2018).

Although its glycosylation function was confirmed, *UGT85A84* transcript levels did not exhibit synergistic change with the glycoside accumulation patterns during flowering (**Figure 3**, **Figure S8A**). This inconsistency may be explained as follows: firstly, UGT enzymes could catalyze a broad range of substrates (Friedericke et al., 2014b). For example, CsGT1 from *C. sinensis* is active towards a variety of substrates, including geraniol (100%), eugenol (84%), (Z)-3-hexenol (62%), benzyl alcohol (48%), 2-PE (9.2%), and linalool (1.4%) (Shoji et al., 2015). Secondly, due to the high stability derived from sugar conjugation, glycosylated terpenoids are less likely to undergo further modifications, thus they are prone to accumulation regardless of the variation of UGTs (Friedericke et al., 2014a). Thirdly, the transcripts of UGTs might accumulate before enzyme accumulation, thereby leading to nonsynchronous changes between transcript level and glycoside content. Moreover, MEP pathway genes and linalool synthase genes are important for linalool formation and conversion. The expression of upstream MEP pathway genes showed a significant increase from the initial to the full blossoming stage, which was consistent with the rapid increase in glycosides of linalool and its oxides. In addition, OfLIS1 and OfLIS2, which directly catalyzed the formation of linalool, exhibited high transcript levels at the late blossoming stage, suggesting they might facilitate the formation of corresponding glycosides (Zeng et al., 2016). Finally, transcriptional control and posttranslational modification may lead to the lack of close correlation between the glycoside levels and the expression of *UGT85A84*, as has been reported for other flowers and fruits. For example, in the grape berry exocarp, the amount of geranyl and neryl glycosides were reported to increase continuously from week 6 to week 17, whereas the mRNA levels of *VvGT7* fluctuated with two peaks during development (Friedericke et al., 2014b).

Linalool and its oxides are essential characteristic aroma-active compounds in *O. fragrans* flowers, which are often glycosylated by UGTs and continuously accumulated during blossoming. This conversion not only directly reduces their emission, but also leads to the storage of glycosylated terpenoids as potential contributors to the aroma of industrial products. This study demonstrated that UGT85A84 is capable of glycosylating linalool and its oxides both *in planta* and *in vitro* with UDP-Glc as the specific sugar donor. Although previous studies have unraveled some terpenoid UGTs responsible for fruit and tea leave aroma (Song et al., 2018), little has been known about the important role of UGTs in regulating the aroma qualities of ornamental flowers that were widely applied in industry. Our findings provide an insight into the molecular mechanism underlying the glycosylation of aroma compounds in flowers and offer new information for manipulating the aroma quality of flowers in future.

## Data Availability Statement

The datasets supporting the conclusions of this study can be found in NCBI using accession number PRJNA564449 (https://www.ncbi.nlm.nih.gov/bioproject/PRJNA564449).

## Author Contributions

RZ and CW conceived and designed the research. RZ conducted the transcriptome sequencing and analyzed the candidate OfUGTs. SH, ZZ, and YW investigated the enzyme functions *in vitro* and *in planta*, respectively. WaX and WeX determined the gene expressions and aroma compound contents. XQ, LZ, and QF provided technical guidance of LC-MS examination. All authors took part in analyzing the data and approved the manuscript.

## Funding

This research was supported by the Fundamental Research Fund for the Central Universities (Project No. 2013PY088).

## Conflict of Interest

The authors declare that the research was conducted in the absence of any commercial or financial relationships that could be construed as a potential conflict of interest.
