# Supplementary material for: UGT85A84 Catalyzes the Glycosylation of Aromatic Monoterpenes in Osmanthus fragrans Lour. Flowers
Source: Front Plant Sci. 2019 Nov 26;10:1376. doi: 10.3389/fpls.2019.01376 (PMC6902048; doi:10.3389/fpls.2019.01376)
Supplement: Supplementary file 1 [file DataSheet_1.pdf]

Supplementary Material

UGT85A84 catalyzes the glycosylation of aromatic monoterpenes in *Osmanthus fragrans* Lour. flowers

Riru Zheng[1,2]*, Zhenyin Zhu[1,2], Yanli Wang[1,2] Shiyang Hu[1,2], Wan Xi[1,2], , Wei Xiao[1,2], Xiaolu Qu[1,2], Linlin Zhong[1,2], Qiang Fu[1,2], Caiyun Wang[1,2]*

*** Corresponding author.**

Caiyun Wang

Email: wangcy@mail.hzau.edu.cn

Supplementary Figures and Tables

Supplementary Figure 1. GO classification of assembled unigenes

Supplementary Figure 2. KOG classification of assembled unigenes

Supplementary Figure 3. KEGG classification of assembled unigenes

Supplementary Figure 4. Analysis of differentially expressed genes (DEGs) between different blossoming stages

Supplementary Figure 5. Alignment of amino acid sequences of UGTs

Supplementary Figure 6. SDS-PAGE analysis of protein UGT85A84

Supplementary Figure 7. LC-MS analysis of sugar donors and aglycones

Supplementary Figure 8. Glycosylation of linalool and its oxides in *Osmanthus fragrans* flowers and its potential function.

Supplementary Table 1. Quality of transcriptome sequencing

Supplementary Table 2. Gene primers used for qRT-PCR analysis of four candidate *UGTs*

Supplementary Table 3. Functional annotation of the transcriptome sequences

Supplementary Table 4. GenBank accession numbers used for construction of the phylogenetic tree in Figure 1

Supplementary Table 5. The correlation between transcript levels of MEP pathway genes and the contents of volatile, free and glycosylated aroma compounds

Supplementary Table 6. K_m of UGTs from different plants

Table S1. Gene primers used for qRT-PCR analysis of four candidate *UGTs*

Primer	Sequence

Actin-F	5'-ATTAGTCCTCTTCCAGCCTTCTTTG-3'
Actin-R	5'-ATTATTTCCTTGCTCATACGGTCAG-3'
*OfU*GT85A82-F0	5'-AAGCCACCAAAGAAAGACG-3'
*OfU*GT85A82-R0	5'-GCACTCCACTGCCAATACTC-3'
*OfU*GT85A83-F0	5'-TTCGCAATGGAGGAGACTGA-3'
*OfU*GT85A83-R0	5'-AGGACCAATGCCGTAAACTG-3'
*OfU*GT85AF3-F0	5'-CGAGCGAAGACTCAAATCC-3'
*OfU*GT85AF3-R0	5'-CAGGAGACAGTGGCGTGAT-3'
*OfU*GT85A84-F0	5'-CTTGCTTGGCACCTTTCTG-3'
*OfU*GT85A84-R0	5'-ACCATTTGTTACCTGGCTCAT-3'

Table S2. Quality of transcriptome sequencing

Sample	Raw reads	Clean reads	Clean bases	Error(%)	Q20(%)	Q30(%)	GC(%)
S1-1	55894680	54270372	8.14G	0.01	97.46	93.31	43.86
S1-2	59656098	57908214	8.69G	0.01	97.38	93.13	44.36
S1-3	50600770	49428240	7.41G	0.01	97.55	93.43	44.01
S2-1	52328672	50804512	7.62G	0.02	97.3	92.96	43.56
S2-2	45858390	44510034	6.68G	0.02	97.26	92.87	43.74
S2-3	45792884	44666850	6.7G	0.01	97.41	93.16	44.16
S3-1	57418394	55655706	8.35G	0.02	97.19	92.74	44.73
S3-2	59510806	57524456	8.63G	0.01	97.73	94.11	45.02
S3-3	55998928	46752178	7.01G	0.01	97.62	93.72	43.19
S4-1	50486756	54124672	8.12G	0.01	97.88	94.41	44.34
S4-2	50486756	49351558	7.4G	0.01	97.54	93.43	43.67
S4-3	49707720	48051954	7.21G	0.01	97.5	93.48	45.3

[1]1, 2 and 3 represent three independent biological replicates

[2]Q20: The percentage of bases with a Phred value >20

[3]Q30: The percentage of bases with a Phred value >30

Table S3. Functional annotation of the transcriptome sequences

Databases	Number of unigenes	Percentage(%)

Annotated in NR	94927	65.83
Annotated in NT	70565	48.94
Annotated in KO	38916	26.99
Annotated in SwissProt	72330	50.16
Annotated in PFAM	65823	45.65
Annotated in GO	66202	45.91
Annotated in KOG	10056	27.78
Annotated in all databases	20095	13.93
Annotated in at least one database	102789	71.28
Total unigenes	144186	100

Table S4. GenBank information and glycosylation substrates of plant UGTs used for construction of sequence alignment and phylogenetic tree

Abbreviation	Plant species	Substrates	Nucleotide ID	Protein ID
UGT85A1	*Arabidopsis thaliana*	Monoterpenes		AAF18537
UGT85A2	*Arabidopsis thaliana*	Citronellol, geraniol, perillyl alcohol	AB016819	BAA34687
UGT85A5	*Arabidopsis thaliana*	Citronellol, geraniol	AC068562	AAF87255
UGT85A7	*Arabidopsis thaliana*	Terpineol, citronellol, geraniol, perillyl alcohol	AC068562	AAF87257
UGT85A23	*Catharanthus roseus*	7-Deoxyloganetin	AB591741	BAK55749
UGT85A57	*Rubus suavissimus*	Diterpenoid	MG592709	
UGT85AF5	*Handroanthus impetiginosus*			PIN05078.1
UGT85AF6	*Sesamum indicum*	7-Deoxyloganetin	XM_011089600.2	XP_011087902.1
UGT85AF7	*Olea europaea*	7-Deoxyloganetin	XM_023005455.1	XP_022861223

Table S5. The correlation between transcript levels of MEP pathway genes and the contents of volatile, free and glycosylated aroma compounds

Genes	Volatile linalool	Free linalool and	Glycosylated linalool

	and its oxides	its oxides	and its oxides
CMK-1	-0.70	0.18	0.80
CMK-2	-0.66	0.27	0.75
DXR	-0.70	0.27	0.82
DXS-1	-0.74	0.12	0.72
DXS-2	-0.61	0.29	0.73
GPPS	-0.58	0.11	0.70
HDS	-0.61	0.31	0.73
IDI	-0.70	0.19	0.77
IDS-1	-0.52	0.37	0.67
IDS-2	-0.55	0.39	0.68
IDS-3	-0.57	0.21	0.68
MCT-1	-0.72	0.20	0.82
MCT-2	-0.64	0.29	0.75
MECPS	-0.60	0.34	0.70
LIS-1	-0.60	-0.11	0.59
LIS-2	-0.51	-0.05	-0.29
LIS-3	0.32	0.31	0.36

[1]The relative coefficient was analyzed according the circadian transcript levels and aroma compounds during the full blossoming period of *O. fragrans* flowers.

Table S6. K_m of UGTs from different plants

Plant	Substrate	K_m(μM)
Vitis vinifera (Friedericke et al., 2014a; Friedericke et al., 2014b)	Nerol	417±4
	Citronellol	433±62
	Geraniol	464±56
	Nerol	204±27
	Citronellol	306±75
	Geraniol	396±12
	Nerol	211±16
	Citronellol	445±21

	Geraniol	321±2
	Nerol	40±3.7
	S-Citronellol	55±1.3
	Geraniol	81±1.0
	8-Hydroxylinalool	33±1.8
	Nerol	118±4.7
	Citronellol	108±2.5
	Geraniol	355±14
Camellia sinensis (Shoji et al., 2015)	Geraniol	44.2±3
	Geranyl β-D-glucopyranoside	78.1±19.6
Prunus persica (Wu et al., 2018)	Linalool	463±80
Actinidia deliciosa (Yar-Khing et al., 2014)	Geraniol	76.2±11.1
	Octan-3-ol	66.6±16.2
	Hexanol	116.9±28.1
