# Supplementary figures and images for: UGT85A84 Catalyzes the Glycosylation of Aromatic Monoterpenes in Osmanthus fragrans Lour. Flowers
Source: Front Plant Sci. 2019 Nov 26;10:1376. doi: 10.3389/fpls.2019.01376 (PMC6902048; doi:10.3389/fpls.2019.01376)

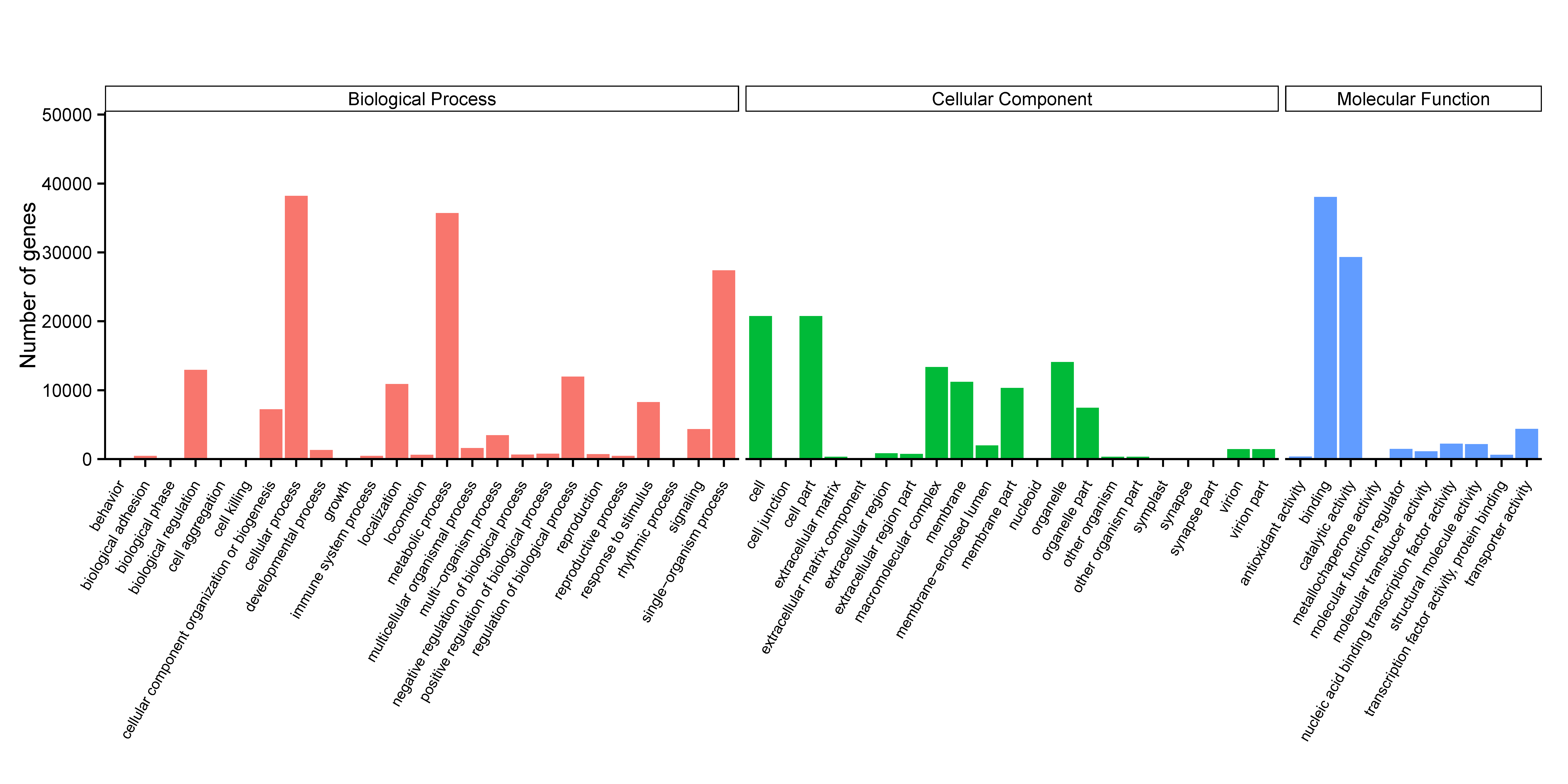

Supplement: Supplementary file 2 [file Image_1.tiff]

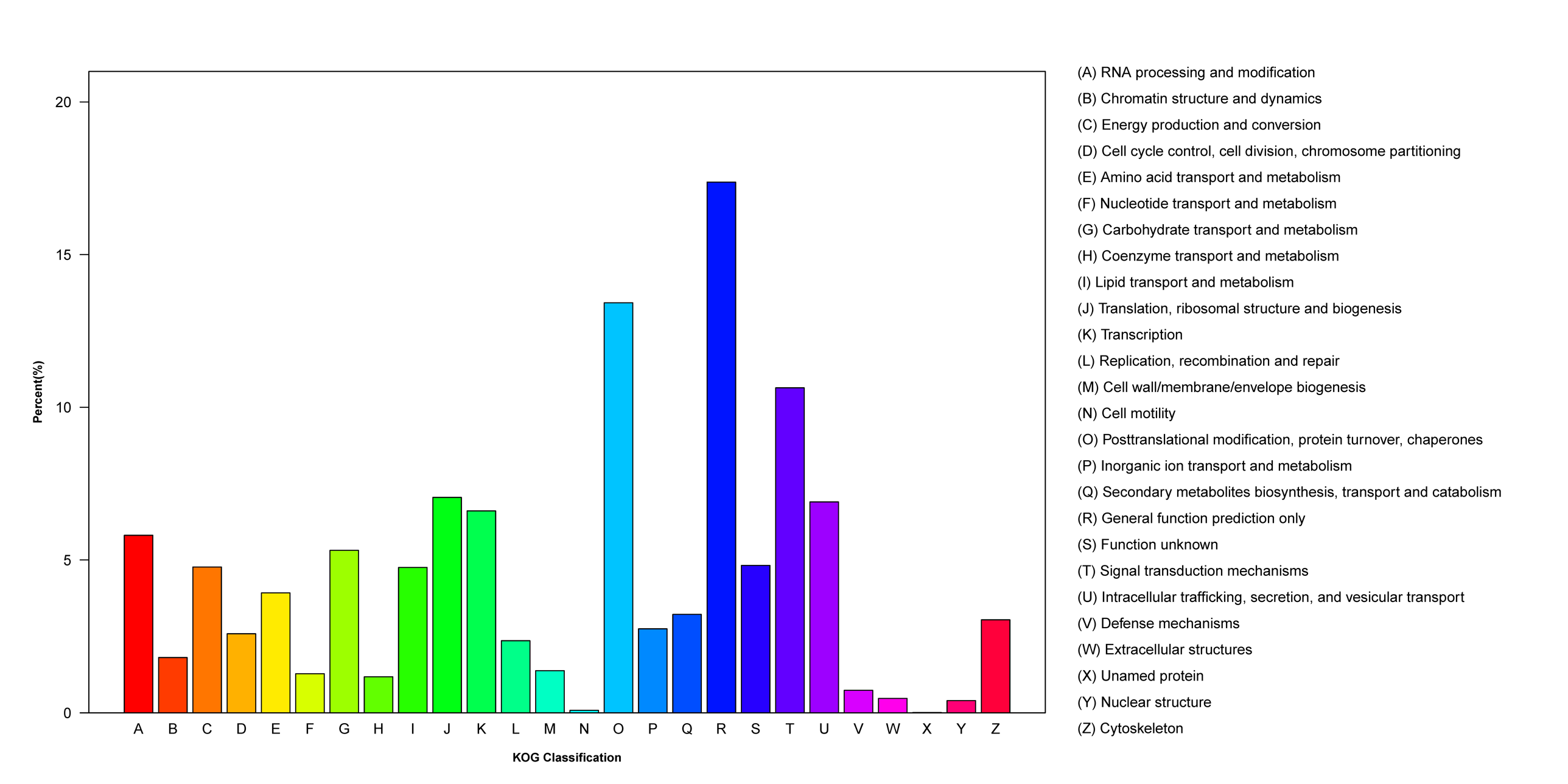

Supplement: Supplementary file 3 [file Image_2.tiff]

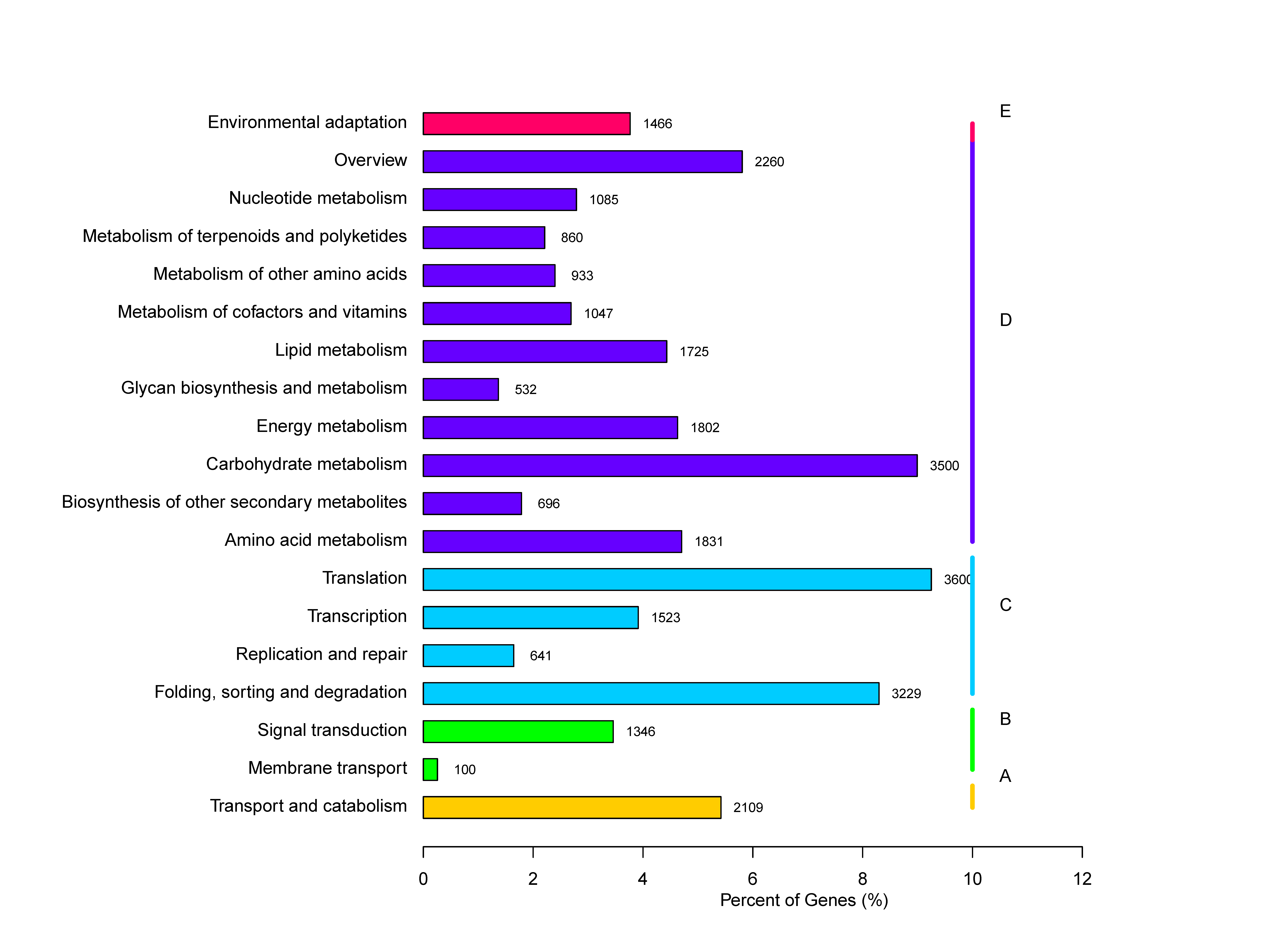

Supplement: Supplementary file 4 [file Image_3.tiff]

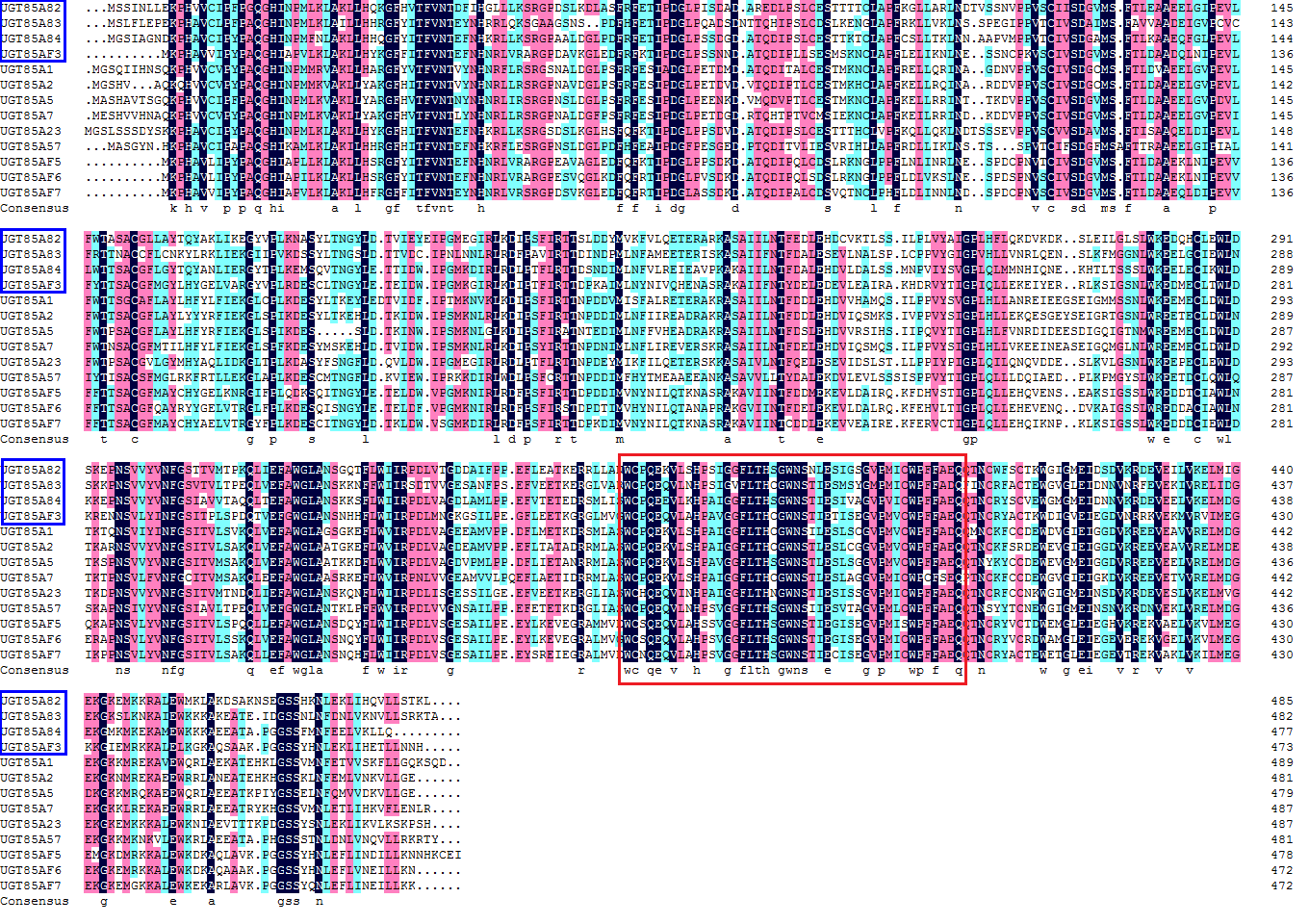

Supplement: Supplementary file 6 [file Image_5.tif]

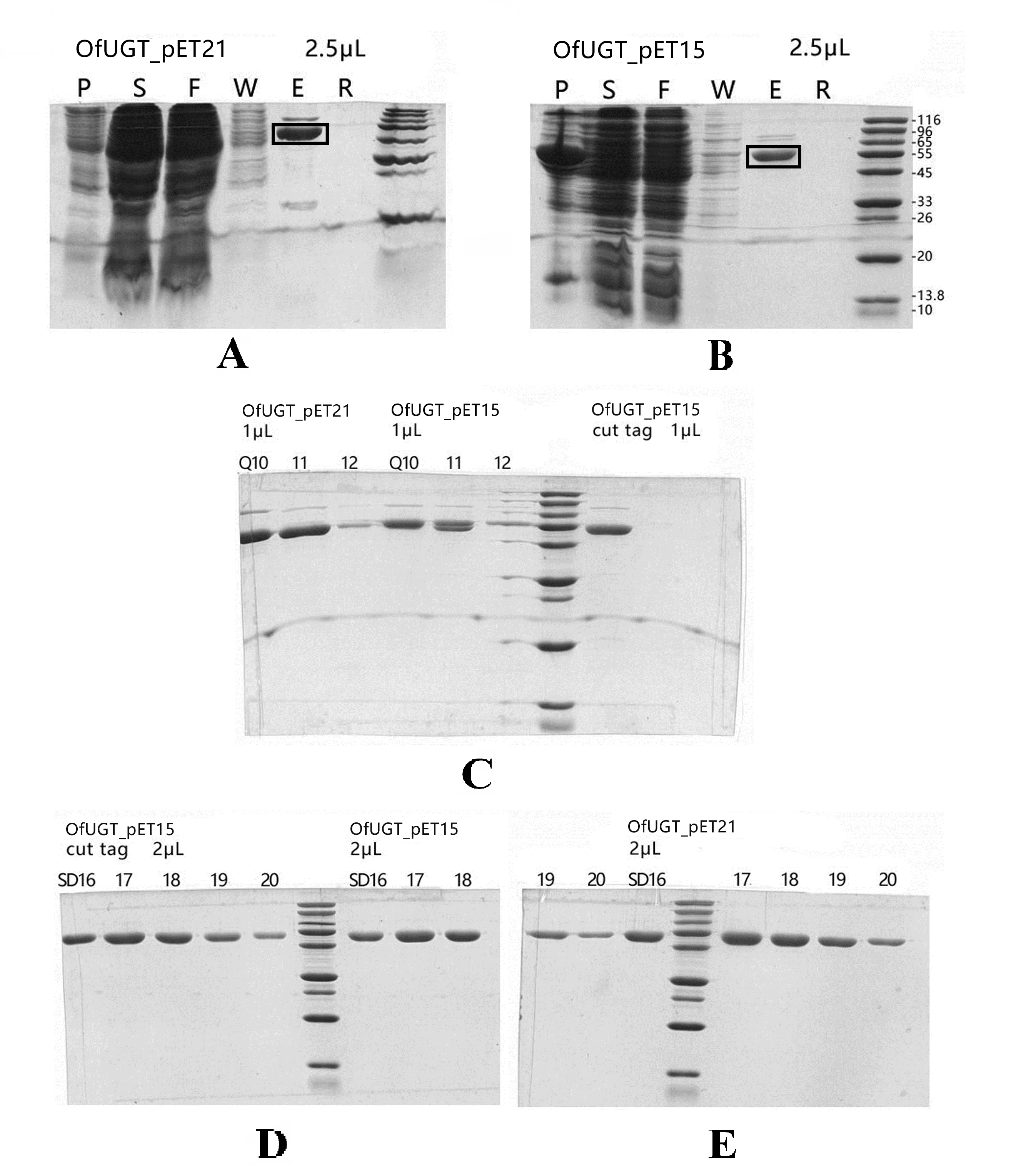

Supplement: Supplementary file 7 [file Image_6.tif]

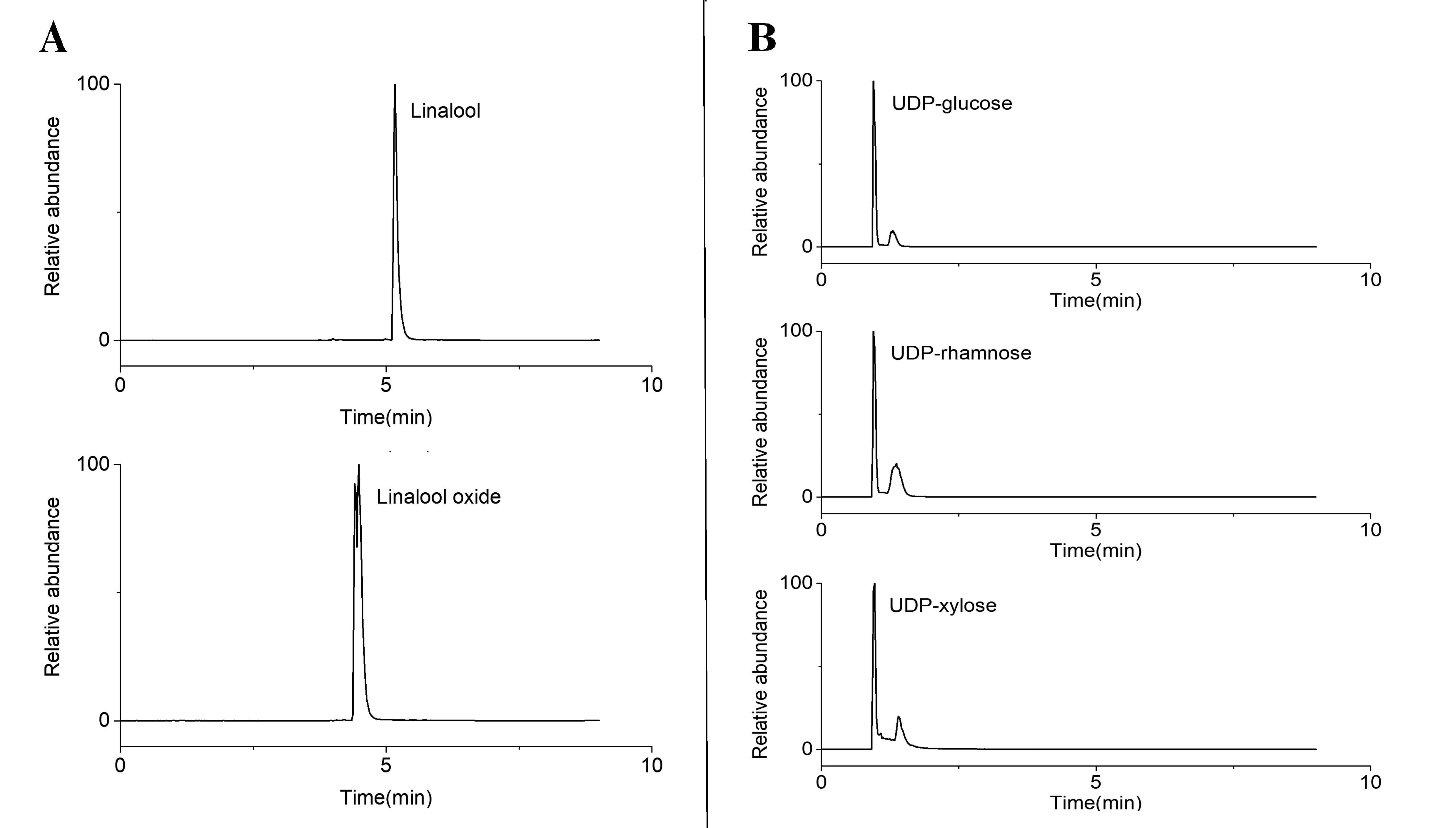

Supplement: Supplementary file 8 [file Image_7.tif]
